# Evidence of depolarization and ellipticity of high harmonics driven by ultrashort bichromatic circularly polarized fields

**DOI:** 10.1038/s41467-018-07151-8

**Published:** 2018-11-09

**Authors:** Lou Barreau, Kévin Veyrinas, Vincent Gruson, Sébastien J. Weber, Thierry Auguste, Jean-François Hergott, Fabien Lepetit, Bertrand Carré, Jean-Christophe Houver, Danielle Dowek, Pascal Salières

**Affiliations:** 1 LIDYL, CEA, CNRS, Université Paris-Saclay, CEA Saclay, 91191 Gif-sur-Yvette, France; 2 Institut des Sciences Moléculaires d’Orsay (ISMO), CNRS, Univ. Paris-Sud, Université Paris-Saclay, F-91405 Orsay, France; 3 CEMES, UPR 8011, CNRS-Université de Toulouse, 29, rue Jeanne Marvig, BP 94347, F-31055 Toulouse, France

## Abstract

High harmonics generated by counter-rotating laser fields at the fundamental and second harmonic frequencies have raised important interest as a table-top source of circularly polarized ultrashort extreme-ultraviolet light. However, this emission has not yet been fully characterized: in particular it was assumed to be fully polarized, leading to an uncertainty on the effective harmonic ellipticity. Here we show, through simulations, that ultrashort driving fields and ultrafast medium ionization lead to a breaking of the dynamical symmetry of the interaction, and consequently to deviations from perfectly circular and fully polarized harmonics, already at the single-atom level. We perform the complete experimental characterization of the polarization state of high harmonics generated along that scheme, giving direct access to the ellipticity absolute value and sign, as well as the degree of polarization of individual harmonic orders. This study allows defining optimal generation conditions of fully circularly polarized harmonics for advanced studies of ultrafast dichroisms.

## Abstract (web summary)

Polarization parameters of the high harmonics driven by bichromatic circularly polarized pulses are usually assumed near perfect. Here the authors use polarimetry measurement to show that depolarization and ellipticity can arise from symmetry breaking in the ionization of a medium by the ultrashort driving fields.

## Introduction

Circularly-polarized light is a remarkable probe of chirality^1^ and magnetization^2,3^. The advent of ultrashort pulses in the extreme ultraviolet (XUV) and soft X-ray ranges of perfectly known and controllable polarization hold the promise of studying these phenomena on the (sub-)femtosecond natural timescales of electronic dynamics and magnetic interactions^4,5^, with atomic selectivity^6,7^. Remarkable progress has been achieved recently to provide tunable polarization of X-ray free-electron lasers, and to characterize the polarization state by combining different techniques^8,9^. Ultrashort table-top high-harmonic generation (HHG) sources have long been confined to low ellipticities^10–15^ until recently when various schemes were shown to produce a high degree of circularity^4,16–21^. A particular scheme proposed long ago^22,23^ stands out as a good candidate to produce intense circularly-polarized ultrashort XUV pulses and has triggered a lot of theoretical^24–30^ and experimental work^17,18,31,32^. In this scheme, the HHG is driven by two counter-rotating circularly polarized pulses at the fundamental and second harmonic frequencies. In the idealized case of perfectly periodic driving fields and isotropic time-independent medium, the threefold dynamical symmetry imposes the emission of all the odd and even harmonics except the 3*q*, with the 3*q*+1 (3*q*+2) harmonics circularly polarized with the same helicity as the *ω* (2*ω*) driver, respectively^17,18,24,33^.

Nevertheless, the complete measurement of the harmonic polarization state (that is, the simultaneous measurement of all three Stokes parameters^34^ for each order) has not yet been performed, to the best of our knowledge. On the one hand, the degree of linear polarization (related to the normalized Stokes parameters *s*_1_ and *s*_2_) were measured using optical polarimetry^17^. On the other hand, the degree of circular polarization (related to the normalized Stokes parameter *s*_3_) was independently measured, in different generation conditions, using X-ray magnetic circular dichroism^18,32^. In all studies, the harmonic emission was considered fully polarized ($\sqrt {s_1^2 + s_2^2 + s_3^2} = 1$), which allows calculating an apparent ellipticity. If this were not the case, that is, if some depolarization were present, the true ellipticity of the polarized part of the radiation would be different. Actually, very few articles have discussed possible causes of depolarization in HHG so far, only in the single color case, and with elliptical driver^13^ or generation in clusters^35^.

The recent theoretical and experimental effort has concentrated on the control of the intensity ratio between the 3*q*+1 and the 3*q*+2 orders^30^, using phase matching^18,36^, the electronic structure of the generation medium^28,31,37,38^, the intensity of the driving fields^39^, or, in a very early study, a strong magnetic field^40^. This ratio is important for determining the polarization of the attosecond pulse train (APT) composed of all harmonic orders. Obviously, the exact polarization of each order is another key element that needs to be determined.

Here we numerically and experimentally show that high harmonics generated by ultrashort counter-rotating *ω* and 2*ω* fields are in general neither purely circular nor fully polarized. In contrast to the usual picture of momentum conservation laws^17,26,27^, time-dependent Schrödinger equation (TDSE) calculations show that elliptical harmonics can already appear at the single-atom level, even in the case of perfectly circular driving fields and isotropic generating medium, resulting from short-pulse envelope effects or fast ionization. Significant deviations from circularity and depolarization are furthermore evidenced by a complete experimental characterization of the polarization state of high harmonics generated in argon, performed with the molecular polarimetry method^41,42^. The molecular frame photoelectron angular distributions (MFPAD) characterizing photoionization of NO molecules provide simultaneously all three Stokes parameters of the XUV light—including the challenging disentanglement of the circular and unpolarized components.

## Results

### Breaking of the dynamical symmetry

**Fig. 1:**
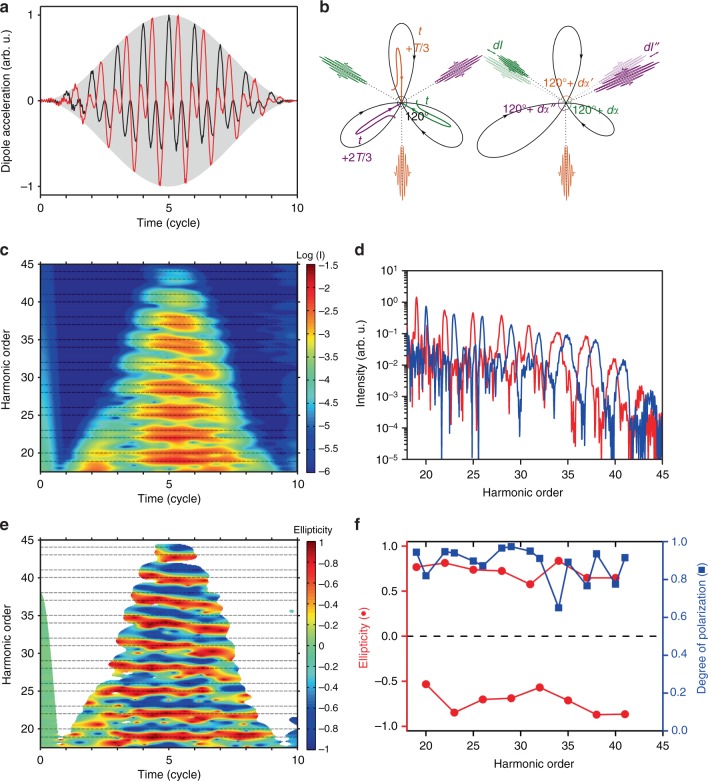
Short-pulse envelope effects in helium. **a** Dipole acceleration along the *x* and *y* components resulting from the interaction of a helium atom with 800- and 400-nm pulses (sin² envelope with 5 *ω*-cycle full-width-at-half-maximum (FWHM) duration, shaded gray) at *I*_*ω*_ = *I*_2*ω*_ = 2 × 10^14^ W/cm². **b** Illustration of the harmonic generation process for a constant laser envelope (left) and on a short-pulse leading edge (right). Typical electron trajectories^24^ launched every *T*/3 of the total driving field (black) are shown in lines of different colors together with the corresponding attosecond pulses emitted at recollision. **c** Time-frequency representation of the harmonic intensity, in logarithmic scale, obtained after Gabor transformation of the dipole shown in (**a**) with a temporal Gaussian window of one-*ω*-cycle FWHM. **d** Corresponding harmonic spectra for the components co-rotating (red) or counter-rotating (blue) with the *ω* field. **e** Time-frequency representation of the harmonic ellipticity, showing the alternating helicity of the 3*q*+1 and 3*q*+2 harmonics. **f** Ellipticity and degree of polarization calculated over the harmonic spectral width (+/−0.25 order until H32 and +/−0.5 order for higher energies)

The combined electric field of the counter-rotating *ω* and 2*ω* beams has a threefold rosette shape (Fig. 1b). The electron wavepackets released through tunnel ionization of the target gas close to each field maximum are accelerated and finally recollide with the parent ion every 1/3 of *ω* cycle^24^. In an isotropic and time-independent medium, this leads to the emission of attosecond pulses of equal intensity every *T*/3, linearly polarized (along the recollision direction) with an orientation increasing by 120° every *T*/3^43^. This threefold dynamical symmetry imposes emission of only the 3*q*+1 and 3*q*+2 harmonic orders with circular polarization and helicity of the *ω* and 2*ω* fields, respectively^18,24,33^. Any breaking of symmetry between the three atto-pulses in the *ω* cycle would result in a decreased harmonic ellipticity, emission of the 3*q* harmonic orders and possibly depolarization. Such breaking can be induced either by the driving fields (elliptical polarization^24,32^ or imperfect overlap^44^) or by the anisotropic generating medium^31,45^. By solving the TDSE, we show that such breaking can occur even for perfectly circular driving fields and in an isotropic medium, when the strong-field interaction leading to the harmonic emission presents sub-*ω* cycle modulations due to (i) a fast temporal variation of the driving laser fields on the envelope rising and falling edges, causing temporal variations of the harmonic dipole vector; (ii) ionization of the medium resulting in a fast decay of the induced dipole strength with time.

The above sources of temporal asymmetry result in a spectral broadening of each harmonic peak with spectrally-varying polarization properties. Ultrafast dichroism measurements involve the polarization characteristics of the short harmonic temporal pulses, that is, the ones calculated over the spectrally-integrated harmonic peaks. The temporal/spectral variations of the polarization may result in a significant amount of depolarization.

A first temporal asymmetry, introduced by the fast variation of the driving fields on the pulse edges, results in a modification of the quantum trajectories leading to the harmonic emission. The recollision time, direction and probability that respectively influence the phase, polarization and intensity of the attosecond emission deviate rapidly (within a *ω* cycle) from the constant-envelope case (by *dt*, *dα*, and *dI*, respectively in Fig. 1b). These variations decrease the ellipticity and polarization degree of the harmonic peaks, as evidenced by TDSE calculations in helium at intensities low enough to avoid significant (<2%) depletion of the medium due to ionization (Fig. 1). The harmonic spectra (Fig. 1d) show well-defined peaks at low orders due to the short electron trajectories (travel time shorter than 0.5*T*) giving the dominant contributions to the harmonic emission^24^. However, the peaks broaden considerably with increasing order leading to an overlap of neighboring orders above H32 and a mixing of their polarization properties. This broadening is a direct consequence of the inter-cycle temporal modulation of the harmonic dipole phase in the laser envelope^46–49^. The resulting harmonic chirp^50–54^ leads to a blue-shift of the central frequency on the rising edge and a red-shift on the falling edge, as evidenced by a Gabor time-frequency analysis of the harmonic intensity (Fig. 1c) and ellipticity (Fig. 1e). For the plateau harmonics (H19-31), the Gabor spectrograms reveal a roughly constant central frequency but an ellipticity almost opposite on the pulse edges as compared to the central part, due to the fast intra-cycle variations illustrated in Fig. 1b. Consequently, the harmonic ellipticity corresponding to the emission during the whole pulse is significantly reduced for all orders to 0.5–0.8, and the depolarization reaches 5 to 10%, and up to 20% for high orders (Fig. 1f). Note that these short-pulse envelope effects depend on the carrier-envelope phase (CEP) and are still significant for 10-cycle FWHM driving fields (Supplementary Note 4).

**Fig. 2:**
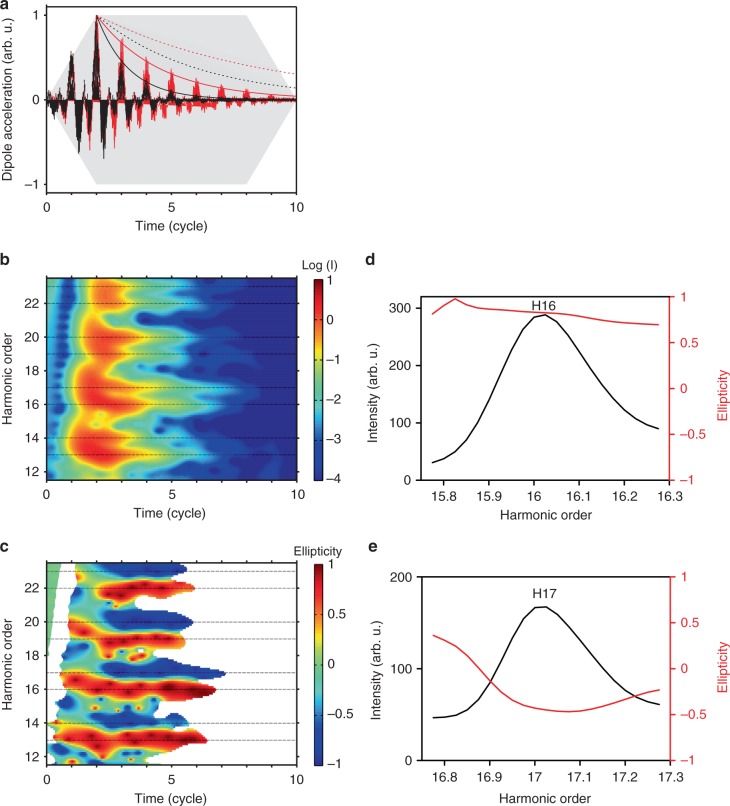
Ionization effects in argon. **a** Dipole acceleration along the *x* axis calculated for trapezoidal pulses (shaded gray area) in argon at intensities *I*_0_ = *I*_*ω*_ = *I*_2*ω*_ = 8 × 10^13^ (red) and 1.2 × 10^14^ W/cm² (black) and their envelopes (exponential solid lines). At 1.2 × 10^14^ W/cm², only the first cycles contribute to the emission due to strong ionization. We show for comparison the dipole envelopes calculated with a linearly-polarized IR pulse of same total energy *I*_*ω*_ = 2*I*_0_ = 1.6 × 10^14^ (red dashed line) and 2.4 × 10^14^ W/cm² (black dotted line). **b** Time-frequency representation of the harmonic intensity, in logarithmic scale, for *I*_0_ = 1.2 × 10^14^ W/cm², obtained after Gabor transformation of the dipole with a temporal Gaussian window of one-*ω-*cycle FWHM. **c** Time-frequency representation of the harmonic ellipticity for *I*_0_ = 1.2 × 10^14^ W/cm². **d**, **e** Spectrum (black) and spectrally-resolved ellipticity (red) of harmonics 16 and 17 for *I*_0_ = 1.2 × 10^14^ W/cm²

**Fig. 3:**
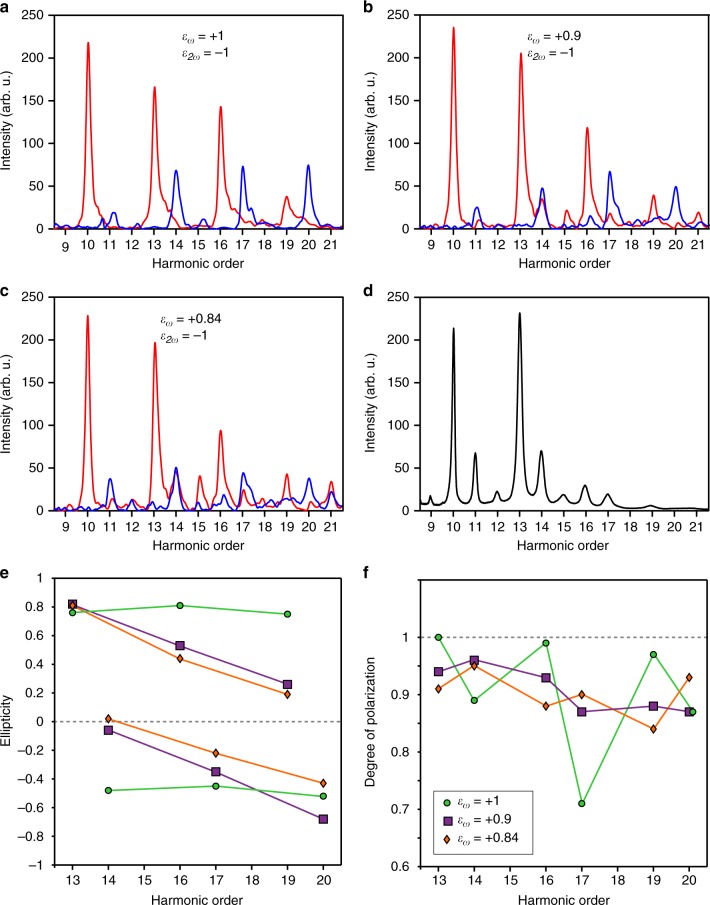
Effects of a slight ellipticity of the *ω* driving field in argon. **a**–**c** High harmonic spectra calculated in argon for trapezoidal pulses of maximal intensity 1.2 × 10^14^ W/cm² and *ω* driver ellipticities *ε*_*ω*_ = +1 (**a**) + 0.9 (**b**) and + 0.84 (**c**), the 2*ω* driver being kept perfectly circular (*ε*_2*ω*_ = −1). The components co-rotating and counter-rotating with the *ω* field are shown in red and blue, respectively. **d** Experimental harmonic spectrum generated in argon in the conditions described in Fig. 4 and measured with the XUV spectrometer. **e**–**f** Ellipticity (**e**) and degree of polarization (**f**) calculated over the harmonic spectral width (+/−0.25 order around the peak), for the 3*q*+1 and 3*q*+2 orders simulated in the plateau region above the ionization potential of argon

A second temporal asymmetry arises from the medium ionization that leads to a decay of the emission efficiency inside the driving envelope. In the bicircular counter-rotating case, the generation medium ionizes much faster than with a linearly polarized field^24^, as illustrated in Fig. 2a for argon. In order to separate this effect from the short-pulse envelope effects discussed above, the simulations are now performed for trapezoidal envelopes (two-cycle turn-on and turn-off and six cycles of constant amplitude), ensuring a constant maximum field strength in the central part of the pulse. There, the dynamical symmetry breaking can only occur in the target, namely through a fast atomic ionization depleting the generating medium. The spectrograms in Fig. 2b, c show two different regions. During the first two cycles, the harmonics are strongly blue-shifted, and their polarization is far from circular. These short-pulse envelope effects are similar to the ones discussed above. During the following cycles, where the envelope is constant, the central frequency and polarization properties are extremely stable with time. However, the circularity is not perfect (|*ε*| ≈ 0.85) due to the exponentially decreasing harmonic efficiency with time, as recovered by a simple model (see Supplementary Note 6). In the corresponding harmonic spectra (Fig. 2d, e), the 3*q*+1 orders exhibit quite constant polarization properties over the harmonic width, in contrast to the 3*q*+2 orders that present much lower and fastly-varying ellipticity, in particular on the low-energy side. This is due to the strong blue-shift of orders 3*q*+1 on the rising edge leading to an overlap with the 3*q*+2. When the ellipticities are calculated over the integrated harmonic peaks, their values deviate significantly from ±1 (|*ε*| ≈ 0.8 for 3*q*+1 and |*ε*| ≈ 0.5 for 3*q*+2) and depolarization appears, mainly for 3*q*+2 orders (10–20%) (Fig. 3e, f).

We now revisit the influence of an additional breaking of symmetry brought about by a slight deviation from circularity of the *ω* field, the 2*ω* field remaining circular. When the *ω* field ellipticity decreases, the 3*q* harmonic intensity increases (Fig. 3a–c), and the harmonic ellipticity quickly drops in modulus for most orders (Fig. 3e)^24^. This has been proposed as a simple method to control the harmonic ellipticity for applications^17^. However, the simulations reveal that, in the studied conditions, the decreased ellipticity goes together with an increased and more homogeneous depolarization (up to 10–15%) (Fig. 3f).

The non-circularity of one driving field may have further consequences if the *ω*–2*ω* relative phase is not controlled in the experiments. Indeed, for significant acquisition times, the polarization characteristics (more precisely, the Stokes parameters) will be a weighted average over all possible relative *ω*–2*ω* phases. In a perfectly circular case, a change in relative phase simply rotates in space the threefold rosette shape of the combined driving fields (see Fig. 1b), which has no influence on the –circular– harmonic polarization^55^. In contrast, with one elliptical driver, in presence of fast ionization or short pulses, this causes a change of the effective electric field over time, and thus of the harmonic polarization characteristics. The simulation of a measurement averaging over twelve different *ω*–2*ω* relative phases in the (*ε*_*ω*_ = +0.9, *ε*_2*ω*_ =−1) case shows a decreased degree of polarization of H17 to only 61% (Supplementary Note 5). Note finally that macroscopic effects can further affect the polarization of the harmonics^18,56^, although they can be minimized by using a thin generating medium such as an effusive jet^31^.

### Complete measurement of the harmonic polarization state

The molecular polarimetry method^41^, recently extended to probe the complete polarization state of a comb of elliptical high-order harmonics^42^, is here applied to the harmonic emission driven in argon by counter-rotating bichromatic fields. This method is based on the anisotropic interaction between an XUV light field and a molecule resulting in photoionization. It consists in measuring MFPAD, a complete angular observable whose expression encapsulates the *s*_1_, *s*_2_, *s*_3_ normalized Stokes parameters of the ionizing light^57,58^. In particular, the *s*_3_ parameter, scaling the intrinsic light helicity, is derived from the molecular frame circular dichroism in the angular distribution (CDAD), the handedness of the system arising from the non-coplanarity of the molecular axis, the photoelectron momentum and the light propagation axis **k**^59^.

**Fig. 4:**
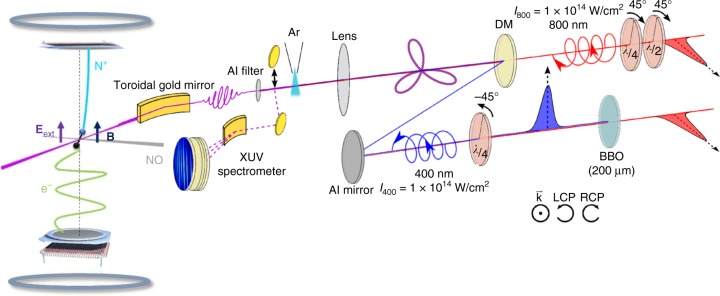
Experimental setup for the complete characterization of the harmonic polarization state. A 1-kHz 3-mJ 800-nm beam is split into two in a Mach–Zehnder interferometer. In one arm, a BBO crystal generates the 400-nm pulses. A zero-order quarter-wave plate (*λ*/4) is placed in each arm to control the ellipticity of the fields, and an additional half-wave plate (*λ*/2) is placed in the fundamental arm to rotate its polarization along the *s* axis. After recombination on a dichroic mirror (DM), the two beams are focused into an argon gas jet for HHG. The XUV emission can be sent to a photon spectrometer with a movable mirror, or filtered with an aluminum foil and refocused by a toroidal mirror at the center of an electron-ion coincidence 3D momentum spectrometer^42, 60^ (and references therein) where it intersects the NO supersonic molecular jet. Electron and ion trajectories are controlled by coupled electric and magnetic fields set in the spectrometer combined with two delay-line time- and position-sensitive detectors, ensuring a 4π collection of both particles for the studied DPI reaction (1). For details see the Methods section

The experiments were conducted on the PLFA beamline (CEA Saclay)^60^ where the XUV pulses generated by the bichromatic field were focused in a COLTRIMS-type reaction microscope^61^ to induce photoionization of randomly oriented NO target molecules (Fig. 4 and Methods section). In order to access MFPADs, we take advantage of prompt dissociative photoionization (DPI) selecting the well characterized prototype reaction^58^:

1 $${\mathrm{NO}}\left( {X^2{\Pi},4\sigma ^25\sigma ^21\pi ^42\pi ^1} \right) + h\nu \to {\mathrm{NO}}^ + \left( {c^3{\Pi},\left( {4\sigma } \right)^{ - 1}} \right) \\ + \, {\mathrm{e}} \to {\mathrm{N}}^ + (\,^3{\mathrm{P}}) + {\mathrm{O}}(\,^3{\mathrm{P}}) + {\mathrm{e}}.$$

The (**V**_N+_,**V**_e_) vector correlation measured for each (N^+^,e) coincident event leads to the electron-ion kinetic energy correlation diagram. The latter enables us to measure the *I*(*θ*_*e*_,*ϕ*_*e*_,*χ*,*γ*) MFPAD corresponding to process (1) for each harmonic peak^42^, where (*θ*_*e*_,*ϕ*_*e*_) represents the electron emission direction in the MF and (*χ*,*γ*) defines the orientation of the molecule in the field frame.

**Fig. 5:**
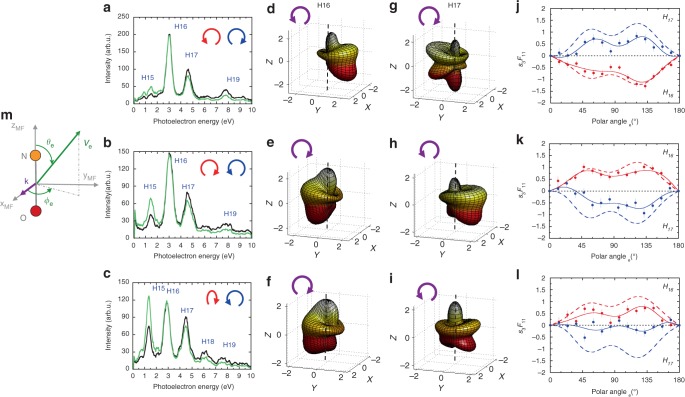
Harmonic helicities characterized by molecular frame photoemission. The three rows correspond to the counter-rotating *ω*–2*ω* driving fields, as labeled in (**a**, **b**, **c**), first row: LCP (*ε*_*ω*_ ≈ −1) and RCP (*ε*_2*ω*_ ≈ +1), second row: RCP (*ε*_*ω*_ ≈ +1) and LCP (*ε*_2*ω*_ ≈ −1), third row: right-elliptically polarized (*ε*_*ω*_ ≈ +0.84) and LCP (*ε*_2*ω*_ ≈ −1). **a**–**c** Photoelectron spectra for reaction (1) induced by the harmonic beam, extracted from the electron-ion kinetic energy correlation diagram as described in ref. ^42^: raw data (black), corrected data accounting for the cross section of reaction (1)^73^ (green). These spectra are discussed in Supplementary Note 7. **d**–**f** H16 and **g**–**i** H17 induced *I*_*χ*=90°_(*θ*_*e*_,*ϕ*_*e*_) MFPADs for reaction (1) with NO oriented perpendicular to the **k** vector as shown in (**m**). **j**–**l** *s*_3_*F*_11_(*θ*_*e*_) (dots and Legendre polynomial line fit) compared to the reference $F_{11}^R(\theta _e)$ functions (dotted line) for photon energies equal to H16 (red) and H17 (blue). $F_{11}^R(\theta _e)$ is displayed with the + or − sign to facilitate comparison with *s*_3_*F*_11_(*θ*_*e*_). All *F*_11_(*θ*_*e*_) functions are normalized relative to the isotropic Legendre component of the MFPAD (see Supplementary Note 1). The error bars correspond to twice the standard deviation resulting from a least squares fit in *χ*, *ϕ*_*e*_*,* and *γ* of the experimental data by the model function (Supplementary Eq. (1))

We first illustrate in the first row of Fig. 5 the determination of the *s*_3_ key parameter for harmonics H16 and H17 generated with left circularly-polarized (LCP, *s*_3,*ω*_ ≈ *ε*_*ω*_ ≈ −1) *ω* field and right circularly-polarized (RCP, *s*_3,2*ω*_ ≈ *ε*_2*ω*_ ≈ +1) 2*ω* field (for H19, see Supplementary Note 2). Figure 5d, g present the two *I*_*χ*=90°_(*θ*_*e*_,*ϕ*_*e*_) MFPADs for NO molecules oriented perpendicular to the **k** vector (averaged on the *γ* angle)—a geometry emblematic of the CDAD effect. The remarkable right–left asymmetries observed in the MFPADs, maximum in the polarization plane (*ϕ*_*e*_ = 90° and 270°), demonstrate the circular character of each harmonic, with helicities of opposite sign for H16 and H17.

These asymmetries are scaled by the *s*_3_*F*_11_(*θ*_*e*_) entangled product^41^, where *F*_11_(*θ*_*e*_) is the photoionization dynamical parameter which governs the CDAD for reaction (1) at each photon energy. The extraction of *s*_3_ for each harmonic peak requires calibrated references $F_{11}^R(\theta _e)$. These were recorded using well-defined polarization states at the DESIRS beamline of SOLEIL synchrotron^62^: $F_{11}^R(\theta _e)$ is positive for such energies, and varies smoothly along the shape resonance of the NO^+^ (*c*^3^*Π*) state^58^. For each harmonic, the *s*_3_ parameter is the affinity coefficient which leads to the best fit between the measured *s*_3_*F*_11_(*θ*_*e*_) and reference $F_{11}^R(\theta _e)$ functions. The derived *s*_3_ parameters amount to −0.8 for H16 and +0.53 for H17 (see Table 1). These values confirm that the helicities of H16 (3*q*+1) and H17 (3*q*+2) possess the sign of the *ω* and 2*ω* helicities, respectively, consistent with XMCD measurements^18,32^. They also evidence their different magnitude, significantly smaller for H17 than for H16^18^. We finally demonstrate in the second row of Fig. 5 that the MFPAD right–left asymmetries and subsequent signs of *s*_3_ are reversed when opposite helicities are used for the *ω* and 2*ω* fields^18^.

**Table 1:** Complete experimental characterization of the harmonic polarization state

800/400	LCP/RCP*ε*_*ω*_ ≈ −1, *ε*_2*ω*_ ≈ +1		RCP/LCP*ε*_*ω*_ ≈ +1, *ε*_2*ω*_ ≈ −1		REP/LCP*ε*_*ω*_ ≈ +0.84, *ε*_2*ω*_ ≈–1		
Harmonic	H16	H17	H16	H17	H15	H16	H17
*s*_1_	0.27 (0.04)	0.32 (0.05)	0.24 (0.04)	0.07 (0.07)	0.13 (0.07)	0.26 (0.05)	−0.24 (0.08)
*s*_2_	0.05 (0.04)	0.03 (0.05)	−0.12 (0.04)	−0.20 (0.07)	−0.13 (0.07)	−0.34 (0.05)	−0.46 (0.07)
*s*_3_	−0.80 (0.04)	0.53 (0.04)	0.77 (0.04)	−0.53 (0.04)	0.33 (0.07)	0.48 (0.04)	−0.22 (0.05)
*s*_4_	0.15 (0.03)	0.38 (0.04)	0.19 (0.04)	0.43 (0.05)	0.62 (0.07)	0.36 (0.04)	0.43 (0.08)
*P*	0.85 (0.03)	0.62 (0.04)	0.81 (0.04)	0.57 (0.05)	0.38 (0.07)	0.64 (0.04)	0.57 (0.08)
*Ψ* (°)	5 (4.5)	2.5 (4.5)	167 (5)	144.5 (9.5)	157.5 (13)	154 (4)	121.5 (4)
*ε*	−0.72 (0.04)	0.56 (0.05)	0.71 (0.04)	−0.68 (0.08)	0.58 (0.12)	0.45 (0.05)	−0.21 (0.04)

Normalized Stokes parameters (*s*_1_, *s*_2_, *s*_3_), depolarization factor *s*_4_, degree of polarization *P*, ellipse orientation *ψ* and ellipticity *ε* for H16 and H17 harmonic peaks for the two configurations of the driving fields: LCP/RCP  ≈  −1/ + 1; RCP/LCP  ≈  + 1/−1; and for H15, H16, and H17 for RCP/LCP ≈ 0.84/−1. Standard deviations are indicated in brackets

The *s*_1_ and *s*_2_ Stokes parameters are extracted from the *I*(*χ*,*γ*) reduced MFPAD after integration over the electron coordinates, that is, the N^+^ ion fragment angular distributions in the field frame^41^ (see Methods and Supplementary Note 1). They evidence here a rather weak degree of linear polarization of the order of 0.2–0.3 (Table 1). Most importantly, measuring coherently the *s*_1_, *s*_2_, *s*_3_ Stokes vector enables us to determine the degree of polarized light $P = \sqrt {s_1^2 + s_2^2 + s_3^2}$, in addition to the geometric parameters of the polarization ellipse, its orientation angle $\psi = \frac{1}{2}{\mathrm{atan}}\left( {\frac{{s_2}}{{s_1}}} \right)$ and signed ellipticity $\varepsilon = {\mathrm{tan}}\left( {\frac{1}{2}{\mathrm{asin}}\left( {\frac{{s_3}}{{\sqrt {s_1^2 + s_2^2 + s_3^2} }}} \right)} \right)$. Their values are summarized in Table 1, where we also feature the degree of depolarization *s*_4_ = 1−*P*. *P* is found to be significantly smaller than 1, with values smaller for H17 (*P* ≈ 0.6) than for H16 (*P* ≈ 0.83). The results for the LCP/RCP and RCP/LCP configurations are remarkably consistent and evidence depolarization in the harmonic emission. The ellipticity values are smaller for H17 than for H16, with |*ε*| ≈ 0.6 and 0.7, respectively. We do not comment on the orientation of the ellipse in a context where the time delay *ω*–2*ω* was not stabilized. The results obtained with bicircular counter-rotating fields thus show deviations from circularity for the H16 and H17 harmonic peaks, their polarization state being described by a large ellipticity (0.6–0.7), a weak degree of linear polarization (0.2–0.3) comparable for both, and an unpolarized component larger for H17 (0.4 versus 0.2).

Finally, the third row in Fig. 5 and the right columns in Table 1 display the results when the *ω* field ellipticity is reduced to *ε*_*ω*_ ≈ + 0.84 (rotation of 5° of the quarter-wave plate) with the 2*ω* field kept quasi-circular (*ε*_2*ω*_ ≈ −1): the harmonic ellipticity is strongly reduced^17^, H16 keeping a higher helicity than H17. The photoelectron spectrum shows a significant increase of H15 (3*q*) intensity, allowing the measurement of an ellipticity of same sign as that of H16, and a high depolarization (Supplementary Note 2). Beyond the statistical uncertainties characterized by the standard deviation (Table 1), a higher accuracy in the polarization state description will benefit from an improved instrumental context (advanced calibration of the optical elements, perfect control of the driving fields CEP and relative phase, …). A major increase of the repetition rate of the driving laser, up to 10 or 100 kHz, will be highly beneficial, since the reported coincidence study at 1 kHz imposed a 2–3 h duration for each measurement.

## Discussion

The reported results provide the complete in situ determination of the polarization state of high harmonics generated by bicircular counter-rotating fields. In particular, the degree of polarization, long assumed to be unity, is here measured. Together with TDSE calculations, the experiments indicate that this scheme may generate non-circular harmonics with polarization characteristics varying spectrally, thus responsible for depolarization. In light of the numerical study, the deviation from circularity and depolarization measured in our experimental conditions partially originate from the fast ionization of the argon generating gas, together with the slight elliptical character of the *ω* driver. These deleterious effects are enhanced by the averaging over multiple *ω*–2*ω* relative phases in the interferometer. Furthermore, short-pulse envelope effects, probably limited in our experiments, may play a significant role at shorter pulse durations as evidenced by simulations.

This study helps defining optimal generation conditions for ultrashort circularly-polarized high harmonics. Short-pulse envelope effects will be minimized by using shaped top-hat, CEP-stable driving laser pulses. The relative time delay between the two pulses should be actively controlled^63^, or passively stabilized by a compact in-line interferometer^64^. Finally, ionization of the generation medium should be avoided, which points toward light rare gases driven by mid-IR driving lasers^32,38^, ensuring a broad harmonic spectrum while keeping low ionization rates. The complete characterization and further optimization of circular harmonic emission will allow advanced studies of chiral-sensitive light–matter interactions such as ultrafast chiral recognition via photoelectron circular dichroism^65–67^, ultrafast magnetization and spin dynamics^5,68,69^.

## Methods

### Experimental setup

Experiments have been performed at the PLFA facility (CEA-Saclay, SLIC) based on a Ti:Sapphire laser delivering 800 nm, 50 fs pulses with 13 mJ energy at 1 kHz repetition rate^60^. The 800 nm beam is separated by a 50/50 beam-splitter at the entrance of a Mach–Zehnder interferometer. In one arm, a BBO crystal (200-µm thick, 31° cut angle) generates the 400 nm, 250 µJ second harmonic pulses. A zero-order quarter-wave plate is placed in each arm to control the ellipticity of the fields, and an additional half-wave plate is placed in the fundamental arm to rotate its polarization along the *s* axis during optimization phases. After recombination on a dichroic mirror, both beams are focused with a lens (*f* = 80 cm) into an argon gas jet to generate an APT mainly composed of 3*q*+1 and 3*q*+2 high-order harmonics (HH). The intensity of the two beams is determined with the cutoff law of the XUV spectrum, when the high harmonics are generated with a single color, linearly polarized. We estimate an equal intensity at focus, *I*_*ω*_ ≈ *I*_2*ω*_ ≈ 1 × 10^14^ W/cm². The relative delay between the two arms is set by a translation stage. Their temporal overlap is determined by maximizing the generation of the harmonics orders produced only when both *ω* and 2*ω* fields are present (that is, even harmonics of the 2*ω* field, here H8, H12, and H16), with linearly-polarized pulses.

With a movable gold mirror, the XUV emission can be sent to a spectrometer composed of a XUV grating, two micro channel plates and a phosphor screen detector and a CCD camera. After optimization and filtering by an aluminum foil (200 nm), the XUV radiation is focused by a gold-coated toroidal mirror (angle of incidence 78.5°, *f* = 60 cm) at the center of the ion-electron coincidence 3D momentum spectrometer CIEL as described in ref. ^42^. Note that the reflection on this mirror modifies the polarization state of the incoming HHs, see Supplementary Note 3. The intersection of the XUV light with the NO molecular beam produced in a supersonic expansion defines the interaction region where photoionization occurs. Ions and electrons are extracted and guided to their respective time- and position-sensitive delay-line detector (TPSD, RoentDek) by coupled electric and magnetic fields, which ensure a 4π collection of both particles for the studied dissociative photoionization (DPI) processes (Eq. (1))^42,60^ and references therein. The ensemble allows for measuring in coincidence the initial 3D velocity vectors of the ion fragment **V**_N+_ and the photoelectron **V**_e_ resulting from the same DPI event. At the 1 kHz repetition rate of the Ti:Sapphire laser, which restricts the total number of coincidences to about 50 c/s, each measurement lasted for a few hours requiring a good stability of the setup.

### Determination of the Stokes parameters for each harmonic

The (*s*_1_,*s*_2_,*s*_3_) normalized Stokes parameters for each HH were determined using the molecular polarimetry method (MP) demonstrated in ref. ^41^. Taking advantage of dissociative photoionization (DPI) of small molecules characterized by electron-ion coincident 3D momentum spectrometry, MP consists in measuring the *I*(*θ*_*e*_,*ϕ*_*e*_,*χ*,*γ*) MFPAD for selected DPI processes, where (*χ*,*γ*) define the polar and azimuthal recoil-ion emission direction in the laboratory frame (LF), and (*θ*_*e*_,*ϕ*_*e*_) the polar and azimuthal photoelectron emission direction in the molecular frame (MF). *I*(*θ*_*e*_,*ϕ*_*e*_,*χ*,*γ*) encodes the (*s*_1_,*s*_2,_*s*_3_) Stokes parameters^58^ of the ionizing light. DPI of the NO molecule into the NO^+^(*c*^3^*Π*) ionic state (see Eq. (1)) is here induced by each peak of the studied harmonic comb^42^. Exploiting the general form of the MFPAD (see Supplementary Eq. (1)) leads to the product *s*_3_ × *F*_11_(*θ*_*e*_) and therefore to the *s*_3_ Stokes parameter, if the *F*_11_(*θ*_*e*_) reference functions characterizing the MF circular dichroism for reaction Eq. (1) at the HH photon energy are determined. The *s*_1_ and *s*_2_ Stokes parameters are derived from the *I*(*χ*,*γ*) LF ion fragment angular distribution after integrating the *I*(*θ*_*e*_,*ϕ*_*e*_,*χ*,*γ*) MFPAD over the electron emission angles. For details see the Supplementary Note 1.

### Theoretical methods

We solve numerically the TDSE in the velocity gauge, on a two-dimensional (2D) Cartesian grid. We first compute the eigenvalues and eigenstates of the stationary Hamiltonian, by means of the imaginary time propagation method, for a soft-core screened Coulomb atomic potential reproducing the ionization potential *I*_p_ of the considered atom (argon *I*_p_ = 0.579 a.u. or helium *I*_p _= 0.903 a.u., where a.u. stands for atomic units), and the 3*p*_*x*_ and 3*p*_*y*_ orbitals for argon:

$$V(x,y) = - \frac{{(Z - 1){\mathrm{e}}^{ - (x^2 + y^2)} + 1}}{{\sqrt {x^2 + y^2 + \eta } }}$$

with *Z* = 18 and *η* = 1.04327 for argon, and *Z* = 2, *η* = 0.67724 for helium. The 3*p*(*m* = ±1) states of argon are constructed from the 3*p*_*x*_ and 3*p*_*y*_ orbitals: 3*p*(*m* = ±1) = 3*p*_*x*_±i3*p*_*y*_. The high laser intensity can mix the degenerate *p* orbitals^70^, so that we do not discuss the relative influence of the co-rotating and counter-rotating orbitals here^28^. The initial wave functions are then propagated forward in time using the split step Fourier method^71^. To avoid spurious effects when the wave packet reaches the boundary of the computational domain, we apply the wavelength-dependent absorption method^72^ at each time step.

The grid size is 819.2 × 819.2 a.u. and the spatial step size is Δ*x* = Δ*y* = 0.2 a.u. The depth of the absorbing layer is 8 a.u. on each side of the grid, along both *x*- and *y*-axis. Note that the grid size was chosen much larger than the maximum wave packet quivering amplitude (∼ 20–30 a.u. in the case of He), and could accommodate for strong ionization without significant loss of wavefunction amplitude. We systematically checked the time evolution of the norm of the wavefunction all along the simulations. The time step is Δ*t* = 6.2 × 10^−2^ a.u. The accuracy is O[(Δ*t*)^3^]. The (relative) spatial frequency resolution is $\frac{{{\Delta k_x}}}{{k_0}}$ = $\frac{{{\Delta k_y}}}{{k_0}}$ = $\frac{{\lambda _0}}{{\Delta x}} = \frac{{\lambda _0}}{{\Delta y}}$ = 7.5 × 10^4^, and the (relative) temporal frequency resolution is $\frac{{\Delta \omega }}{{\omega _0}}$ = $\frac{T}{{\Delta t}}$ =1.6 × 10^3^. This ensures a very accurate description of the relatively low-order harmonics considered in this work.

The laser field is the sum of two counter-rotating circularly polarized waves at *λ*_1_ = 800 nm and *λ*_2_ = 400 nm central wavelengths, respectively:

$${\mathbf{E}}\left( {{t}} \right) = f\left( t \right)\left\{ \begin{array}{l}\left[ E_1\cos \left( \omega _1t + \varphi _{\mathrm{IR}} + \varphi _{\mathrm{CEP}} \right) + E_2\cos \left( \omega _2t + 2\varphi _{\mathrm{CEP}} \right) \right]{\mathbf{e}}_x \\ + \left[ E_1\varepsilon _1\sin \left( \omega _1t + \varphi _{\mathrm{IR}} + \varphi _{\mathrm{CEP}} \right) + E_2\varepsilon _2\,{\mathrm{sin}}\left( \omega _2t + 2\,\varphi _{\mathrm{CEP}} \right) \right]{\mathbf{e}}_y\end{array} \right\}$$

with $\omega _2 = 2 \times \omega _1,\,E_i = \frac{{E_0}}{{\sqrt {1 + \varepsilon _i^2} }}$, and *ε*_*i*_ the field ellipticity, *i* = 1, 2. *φ*_CEP_ is the carrier-envelope phase, acting on both *ω*_1_ and *ω*_2_ fields, whereas *φ*_IR_ is a phenomenological phase term applied only on the IR field for simulating a dephasing between the two fields induced, e.g., by instabilities in the Mach–Zehnder interferometer used to split the beams (see Fig. 4). The pulse envelope *f*(*t*) has a sine square shape with five-*ω*-cycle FWHM in Fig. 1, and a trapezoidal shape with a two-IR cycle turn-on, a six-cycle plateau, and a two-cycle turn-off in Fig. 2, 3. In all the presented figures, *φ*_CEP_ = 0.

High-order harmonic spectra are obtained by Fourier-transforming the time-dependent, space-integrated components of the dipole acceleration. For characterizing the polarization properties of the harmonics, we calculate the Stokes parameters^34^ following the method described in ref. ^13^. The Stokes parameters are further averaged over a spectral width Δ*ω* around the harmonic central frequency Ω according to: $S_i(\Omega ) = {\int}_{\Omega - \frac{{\Delta \omega }}{2}}^{\Omega + \frac{{\Delta \omega }}{2}} {S_i(\omega )\mathrm{d}\omega }$, and the normalized Stokes parameters are calculated following $s_i(\Omega ) = \frac{{S_i(\Omega )}}{{S_0(\Omega )}}$.

We emphasize here that the HHG process being coherent, the emission is fully polarized for each frequency inside the harmonic peak. However, the polarization characteristics may vary spectrally for reasons discussed above. Consequently, the spectrally-integrated emission of a particular harmonic (corresponding to the emission during the whole harmonic temporal profile) may exhibit a reduced degree of polarization.

### Code availability

The custom computer code used in the current study is available from the corresponding authors on reasonable request.

## Electronic supplementary material

Supplementary Information

## Data Availability

The datasets generated during the current study are available from the corresponding authors on reasonable request.
