# Supplementary material for: Evidence of depolarization and ellipticity of high harmonics driven by ultrashort bichromatic circularly polarized fields
Source: Nat Commun. 2018 Nov 9;9:4727. doi: 10.1038/s41467-018-07151-8 (PMC6226473; doi:10.1038/s41467-018-07151-8)
Supplement: Supplementary file 1 — Supplementary Information [file 41467_2018_7151_MOESM1_ESM.pdf]

Supplementary information

Evidence of depolarization and ellipticity of high-harmonics driven by ultrashort bichromatic circularly-polarized fields

Barreau *et al.*

Outline

Supplementary Note 1: Molecular polarimetry: determination of the (s_1, s_2, s_3) Stokes parameters.. 2

Supplementary Note 2: Molecular polarimetry measurements on harmonics H15 and H19 5

Supplementary Note 3: Calibration of the effects of the toroidal mirror on the XUV polarization 5

Supplementary Note 4: Breaking the dynamical symmetry with short pulses...................................... 8

Supplementary Note 5: Influence of the relative phase of the two driving fields................................. 9

Supplementary Note 6 : Simple polarization model in the case of an exponentially decreasing efficiency 10

Supplementary Note 7 : Discussion of the shape of the harmonic spectra... 11

Supplementary references 12

Supplementary Note 1: Molecular polarimetry: determination of the (s_1, s_2, s_3) Stokes parameters

The molecular polarimetry (MP) method relies on the general form of the $I(\theta_e, \phi_e, \chi, \gamma)$ MFPAD, for each dissociative photoionization process characterized by electron-ion coincident 3D momentum spectroscopy [1]. For one-photon ionization of a linear molecule induced by elliptically polarized light, described by the (s_1, s_2, s_3) normalized Stokes parameters [2], it writes in the dipole approximation [1, 3]:

$$
\begin{aligned}
I(\theta_e,\phi_e,\chi,\gamma) = {} & F_{00}(\theta_e) + F_{20}(\theta_e)\left[-\frac{1}{2}P_2^0(\cos\chi) + \frac{1}{4}t_1(\gamma)P_2^2(\cos\chi)\right] \\
& + F_{21}(\theta_e)\left\{\left[-\frac{1}{2} - \frac{1}{2}t_1(\gamma)\right]P_2^1(\cos\chi)\cos(\phi_e) - \frac{3}{2}t_2(\gamma)P_1^1(\cos\chi)\sin(\phi_e)\right\} \\
& + F_{22}(\theta_e)\left\{\left[-\frac{1}{2}P_2^2(\cos\chi) + t_1(\gamma)(2 + P_2^0(\cos\chi))\right]\cos(2\phi_e) + 3t_2(\gamma)P_1^0(\cos\chi)\sin(2\phi_e)\right\} \\
& - s_3F_{11}(\theta_e)P_1^1(\cos\chi)\sin(\phi_e)
\end{aligned}
$$

$$\text{with } t_1(\gamma) = s_1\cos(2\gamma) - s_2\sin(2\gamma) \text{ and } t_2(\gamma) = s_1\sin(2\gamma) + s_2\cos(2\gamma) \quad (1)$$

where P_L^N are the associated Legendre polynomials. Measuring MFPADs therefore enables one to determine the complete polarization state of the ionizing light in terms of the encoded (s_1, s_2, s_3) Stokes parameters [4], as well as the $F_{LN}(\theta_e)$ functions containing all the dynamical information on the photoionization (PI) process, as their partial-wave expansion in a Legendre polynomial basis gives access to the amplitudes and phases of the complex dipole matrix elements of the PI transition [3, 5]. However, since s_3 appears in the product $s_3 \times F_{11}(\theta_e)$ in Supplementary Eq. (1), retrieving its amplitude and sign (helicity) requires the knowledge of a reference $F_{11}(\theta_e)$ function, characterizing the circular dichroism in the MF, from an independent experiment or calculation. Based on this remarkable property, the MP method was demonstrated using synchrotron radiation with well-defined polarization state (DESIRS beamline, SOLEIL) [4]. The benchmarked DPI reaction of the NO(X,$^2\Pi$) molecule corresponding to ionization of the 4σ inner-valence shell molecular orbital, main text Eq.(1) and Supplementary Eq. (2), was chosen since it possesses the fingerprint properties to act as an efficient molecular polarimeter [4].

$$\mathrm{NO}(X\,^2\Pi, 4\sigma^2 5\sigma^2 1\pi^4 2\pi^1) + h\nu \rightarrow \mathrm{NO}^+(c\,^3\Pi, (4\sigma)^{-1}) + \mathrm{e} \rightarrow \mathrm{N}^+(^3P) + \mathrm{O}(^3P) + \mathrm{e} \quad (2)$$

The extraction of the s_3 parameter is discussed in the main text and illustrated in Fig. 5. In Supplementary Fig. 1, we show the measured $s_3 \times F_{11}(\theta_e)$ resulting from DPI of NO by H17 (see legend) and $F_{11}^R(\theta_e)$ from a synchrotron-based experiment used here as a reference [6]. The comparison of the two led to $s_3 = +0.53$ for the considered harmonic. s_3 uncertainties are given by

the standard deviation in the $F_{11}(\theta_e)$ affinity process. For completeness, we also report in Supplementary Fig. 1 the other $F_{00}(\theta_e)$, $F_{20}(\theta_e)$, $F_{21}(\theta_e)$, $F_{22}(\theta_e)$ functions as an example for DPI of NO induced by the selected H17 harmonic (see legend) and synchrotron radiation at the corresponding $h\nu = 26.35$ eV photon energy. The very fair agreement, despite the rather low statistics (≈ 3000 events in the selection) in the HHG experiment ran at the 1 kHz laser rep-rate, emphasizes the potential of the F_{LN}-based data analysis methodology.

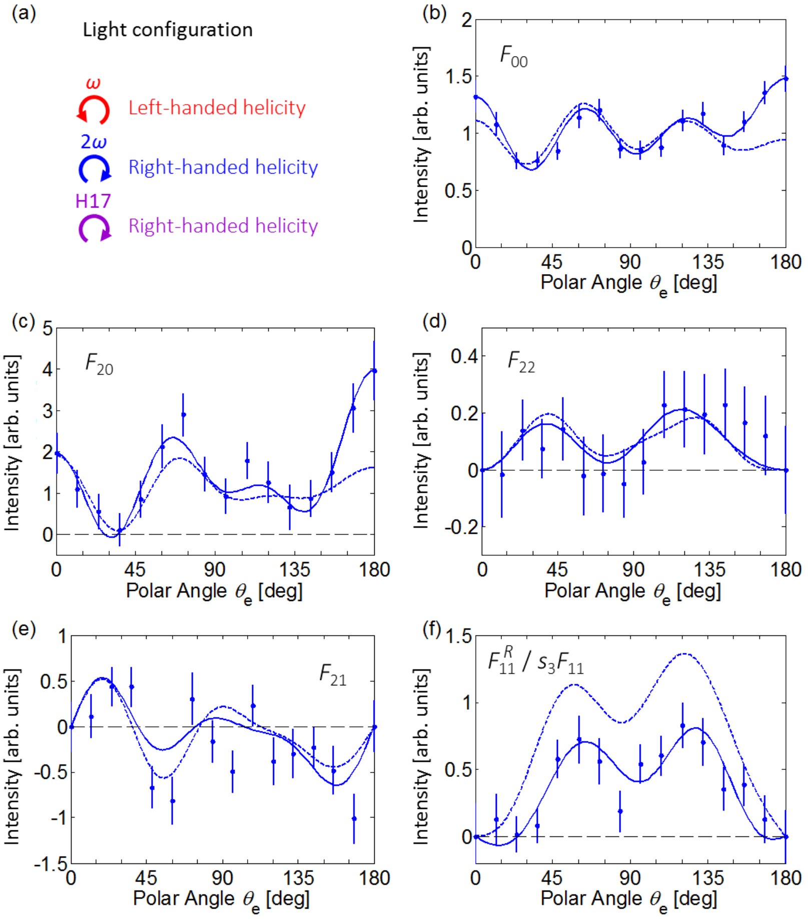

Supplementary Figure 1: Experimentally retrieved $F_{LN}(\theta_e)$ functions. (a) Helicities of the ω and 2ω driving fields and of the produced harmonic H17. Measured (b) $F_{00}(\theta_e)$, (c) $F_{20}(\theta_e)$, (d) $F_{22}(\theta_e)$, (e) $F_{21}(\theta_e)$ and (f) product $s_3 \times F_{11}(\theta_e)$ for DPI of NO induced by the selected H17 harmonic from APTs generated with the $\varepsilon_\omega \approx -1/\varepsilon_{2\omega} \approx +1$ driving fields configuration (a) (dots and full lines), and pure right-handed circularly polarized synchrotron radiation at $h\nu = 26.35$ eV (dashed lines). Full and dashed lines represent a Legendre polynomial fit. The synchrotron-based measurement $F_{11}^R(\theta_e)$ [6] is used here as a reference for the determination of s_3. HHG and synchrotron experiments are normalized such that the mean value of $F_{00}(\theta_e)$ is unity. The error bars correspond to twice the standard deviation of a least squares fit in χ, ϕ_e and γ of the experimental data by the model function (Supplementary Eq. (1)).

The extraction of the s_1 and s_2 Stokes parameters proceeds after integrating the $I(\theta_e, \phi_e, \chi, \gamma)$ MFPAD over the electron emission angles. It reduces to the $I(\chi,\gamma)$ LF ion fragment angular distribution [3, 4]:

$$I(\chi,\gamma) = C\left\{P_0^0(\cos\chi)\left[1+\frac{\beta}{2}\left(s_1\cos(2\gamma)-s_2\sin(2\gamma)\right)\right]-\frac{\beta}{2}P_2^0(\cos\chi)\left[1+s_1\cos(2\gamma)-s_2\sin(2\gamma)\right]\right\} \quad (3)$$

The $I(\chi)$ polar dependence (integrated over γ) provides the β asymmetry parameter. If β is nonzero, the Fourier analysis of the $I(\gamma)$ azimuthal dependence (integrated over χ) then provides s_1 and s_2. Supplementary Fig. 2 presents the measured $I(\chi)$ polar and $I(\gamma)$ azimuthal dependences for DPI of NO induced by the selected H17 harmonic from APTs generated for two different $\varepsilon_\omega / \varepsilon_{2\omega}$ driving fields configurations (see legend). The analysis of $I(\chi)$ ((a) and (c)) leads to a large asymmetry parameter $(\beta \approx +1)$. In (d), the oscillations observed in $I(\gamma)$ are more pronounced than the ones in (b), reflecting higher s_1 and s_2 values. s_1 and s_2 uncertainties are given by the standard deviation of a least squares fit in χ and γ of the experimental data by the model function (Supplementary Eq. (3)).

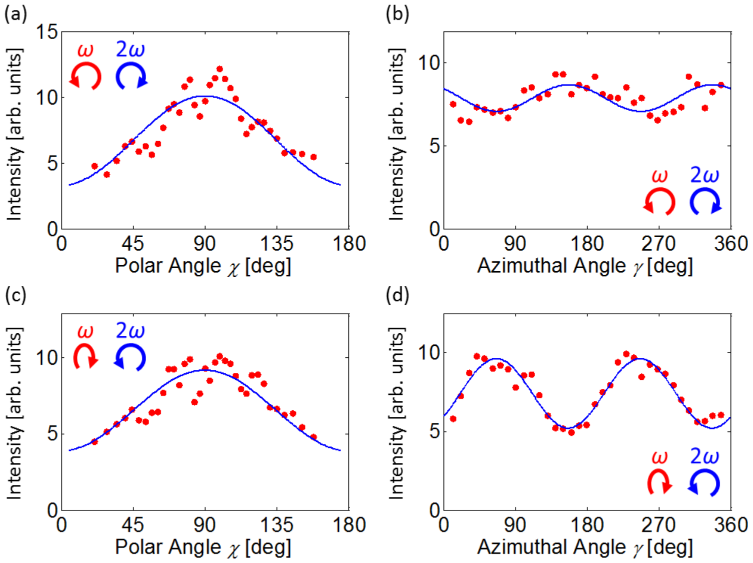

Supplementary Figure 2: Laboratory frame N⁺ angular distribution. Measured (a) $I(\chi)$ polar and (b) $I(\gamma)$ azimuthal dependences of the $I(\chi,\gamma)$ N^+ ion fragment angular distribution in the LF corresponding to DPI of NO induced by the selected H17 harmonic from APTs generated with $\varepsilon_\omega \approx -1 / \varepsilon_{2\omega} \approx +1$ driving fields configuration. (c-d) Same as (a-b) with $\varepsilon_\omega \approx +0.84 / \varepsilon_{2\omega} \approx -1$.

Finally, once the complete set of (s_1, s_2, s_3) Stokes parameters is determined, one can express in an equivalent way the polarization state of the ionizing light in terms of the orientation ψ and signed ellipticity ε of the polarization ellipse, and the degree of polarization P [2].

Supplementary Note 2: Molecular polarimetry measurements on harmonics H15 and H19

In this section, we present additional results obtained with the MP method, characterizing harmonics H15 – which starts building up when one of the ω and 2ω driving fields is not perfectly circularly polarized – and H19 which has a weak contribution in the APT. As displayed in Supplementary Fig. 3, the measured 3D MFPAD and product $s_3 \times F_{11}(\theta_e)$ show that H19 behaves the same way as H16, as predicted for harmonics of the same group (here 3*q*+1) [7]. Besides, when not fully suppressed, H15 (3*q*) exhibits the same helicity as H16 (3*q*+1). This is in agreement with results reported previously for temporally overlapping pulses [7-9].

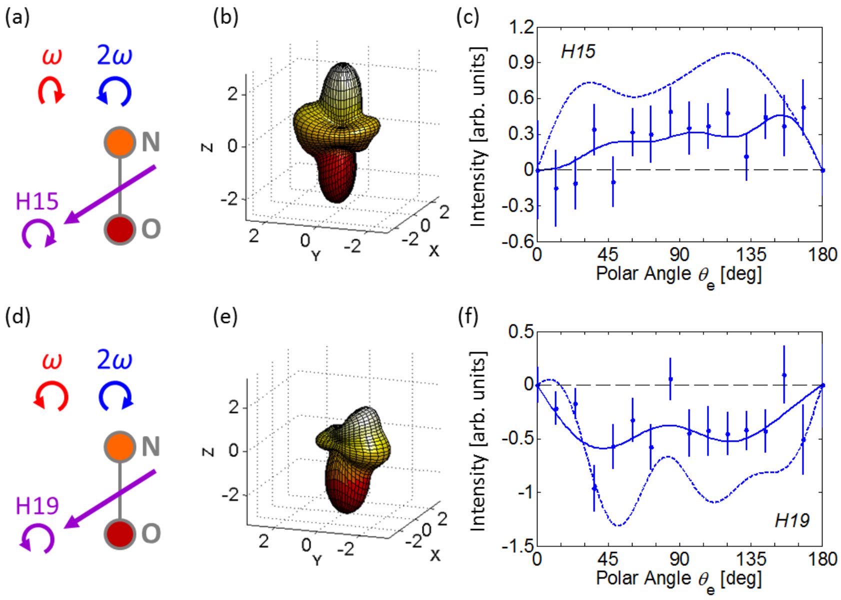

Supplementary Figure 3: **Harmonic helicities characterized by molecular frame photoemission**. (a) Sketch of the $\varepsilon_\omega / \varepsilon_{2\omega}$ configuration, (b) measured 3D MFPAD and (c) product $s_3 \times F_{11}(\theta_e)$ (dots and full lines) for harmonics H15 with $\varepsilon_\omega \approx +0.84 / \varepsilon_{2\omega} \approx -1$. (d-f) Same as (a-c) for H19 with $\varepsilon_\omega \approx -1 / \varepsilon_{2\omega} \approx +1$. In both cases, the dashed line is the corresponding synchrotron-based $F_{11}^R(\theta_e)$ reference [6]. Full and dashed lines represent a Legendre polynomial fit. The error bars correspond to twice the standard deviation of a least squares fit in χ, ϕ_e and γ of the experimental data by the model function (Supplementary Eq. (1)).

Supplementary Note 3: Calibration of the effects of the toroidal mirror on the XUV polarization

The polarization state of the XUV light is modified by the reflection on the gold-coated toroidal mirror (angle of incidence 78.5°, *f* = 60 cm) used to focus the beam into the COLTRIMS interaction region, where it induces photoionization (PI) of a gas phase target. It is thus necessary to fully

characterize this reflection in order to recover the polarization state of the high harmonics composing the XUV attosecond pulse train right after generation. The XUV polarization state is described by the Stokes vectors $S_{\mathrm{XUV,PI}}$ measured by molecular polarimetry [2, 10], and $S_{\mathrm{XUV,HHG}}$ the sought-for polarization state of the HHG emission. The gold mirror is described by a Mueller matrix $\boldsymbol{M}_{\mathrm{Mirror}}$ [11] so that:

$$S_{\mathrm{XUV,PI}} = \boldsymbol{M}_{\mathrm{Mirror}} S_{\mathrm{XUV,HHG}} \tag{4}$$

$$S_{\mathrm{XUV,HHG}} = \boldsymbol{M}_{\mathrm{Mirror}}^{-1} S_{\mathrm{XUV,PI}} \tag{5}$$

and

$$\boldsymbol{M}_{\mathrm{Mirror}} = \frac{1}{2}\begin{bmatrix} R_s + R_p & R_s - R_p & 0 & 0 \\ R_s - R_p & R_s + R_p & 0 & 0 \\ 0 & 0 & 2\sqrt{R_s R_p}\cos\delta & -2\sqrt{R_s R_p}\sin\delta \\ 0 & 0 & 2\sqrt{R_s R_p}\sin\delta & 2\sqrt{R_s R_p}\cos\delta \end{bmatrix} \tag{6}$$

where R_s and R_p are the reflectivities in intensity of the *s*- and *p*-polarized components of the light, respectively, and δ is the dephasing between these components induced by a reflection on the mirror. To determine these parameters for each harmonic, we used molecular polarimetry to measure the (s_1, s_2, s_3) normalized Stokes vector of the reflected light originating from known, controlled, linearly polarized high harmonics generated in SF_6 by a linearly polarized IR field for various polarization angles α in the [-90°, 90°] range, where α is defined relative to the mirror reference axis.

For this calibration study, (i) the s_3 parameter was measured using dissociative photoionization (DPI) of the NO molecule with 45° sampling, providing also s_1 and s_2, and (ii) the s_1 and s_2 parameters were additionally measured with 10° sampling by analyzing the photoelectron angular distribution in the laboratory frame for PI of helium, whose higher count rate allowed faster acquisitions. The values of the $R = R_p/R_s$ and δ parameters characterizing the mirror for harmonics H15 to H21 were successively derived by a fit of the α-dependence of the s_1 (R) and s_3 (δ) quantities, respectively, according to the relations:

$$s_1 = \frac{R - 1 - (R+1)\cos(2\alpha)}{R + 1 - (R-1)\cos(2\alpha)} \tag{7}$$

$$s_3 = \frac{2\sin(2\alpha)\sqrt{R}\sin(\delta)}{R + 1 + (R-1)\cos(2\alpha)} \tag{8}$$

We illustrate the quality of the fits in Supplementary Figs. 4 and 5 which display respectively the evolution of the difference between the measured s_1 parameter for the actual mirror and that of a perfect mirror $e_1 = s_1(R = 1)$, and the measured s_3 as a function of the initial polarization direction α for harmonics 17, 19 and 21.

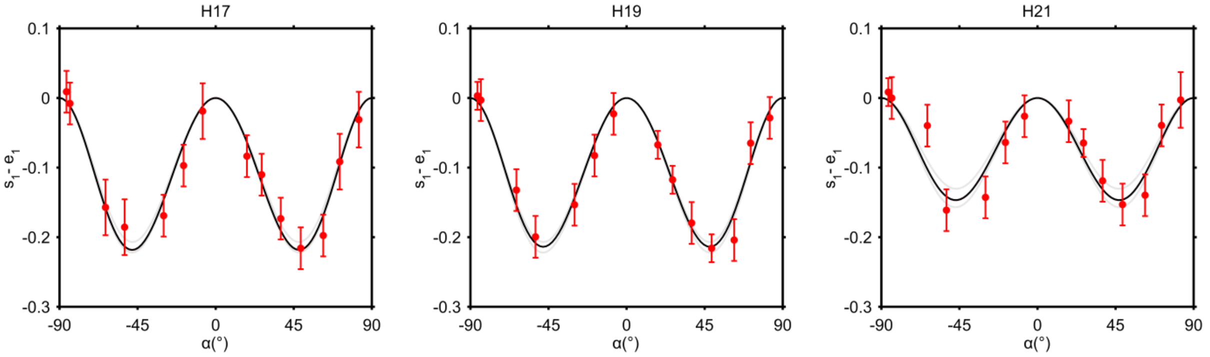

Supplementary Figure 4: Calibration of the mirror response for the s_1 parameter. Difference between the measured s_1 Stokes parameters for the actual mirror and those of a perfect mirror e_1 as a function of the initial polarization direction (red dots) and their least-square fits according to Supplementary Eq. (7) (black line), for harmonics 17, 19 and 21. s_1 error bars correspond to twice the standard deviation.

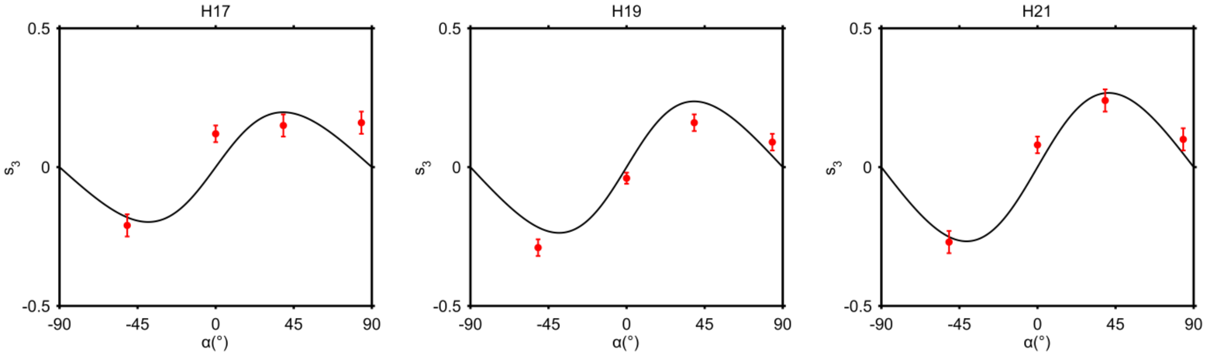

Supplementary Figure 5: **Calibration of the mirror response for the s_3 parameter.** Measured s_3 Stokes parameter as a function of the initial polarization direction (red dots) and their least squares fits according to Supplementary Eq. (8) (black line). s_3 error bars correspond to twice the standard deviation.

Harmonic order	R_{exp}	R_{theo}	δ_{exp}(°)	δ_{theo} (°)
17	0.65±0.01	0.65	-11.4±4.5	-21.3
19	0.65±0.01	0.63	-13.7±3.1	-22.0
21	0.75±0.02	0.69	-15.5±2.5	-21.7

Supplementary Table 1: Mirror reflectivity characteristics. Experimental and theoretical values of the ratio of reflectivities R and dephasing δ between the *s*- and *p*-polarized components of harmonics 17 to 21.

The values of the R and δ parameters extracted from the corresponding fits are summarized in Supplementary Table 1 and compared with those of an ideal gold mirror [11]. The values of the dephasing δ are found of lower magnitude (by less than 10°) than the theoretical values. This difference might originate from a thin organic deposit at the surface of the gold mirror, modifying its refractive index.

This transmission function of the toroidal mirror is used throughout the work presented to derive the HHG reduced Stokes parameters from the values measured after the mirror using molecular polarimetry. For the even harmonics, such as H16, which are not generated with linearly polarized light, the parameters were extrapolated between those for H15 and H17.

Supplementary Note 4: Breaking the dynamical symmetry with short pulses

In the main text Fig. 1, we discuss the deviations from circularity and depolarization calculated for high harmonics generated in helium by 5 ω-cycle FWHM (≈ 13.3 fs) circularly-polarized driving pulses at $I_{\omega}=I_{2\omega}=2 \times 10^{14}$W/cm². The same calculations have been performed for 10 ω-cycle FWHM (≈ 26.6 fs) pulses at the same intensities. The corresponding ellipticity and degree of polarization are displayed in Supplementary Fig. 6. Harmonics 22 to 41 possess a high degree of polarization although not equal to 1 ($P \approx 0.9$). This value is of the order of the degree of polarization found in the main text Fig. 1e for orders lower than 33 that are still narrower than the +/- 0.25 order bandwidth. With 10 ω-cycle pulses, the harmonics' ellipticity is higher (in absolute value) compared to the 5 ω-cycle case. However, deviations from circularity due to the fast-varying envelope are still evidenced with $|\varepsilon| \approx 0.8 - 0.9$ for the $3q$+1 orders and $|\varepsilon| \approx 0.7 - 0.8$ for the $3q$+2, even for these 26.6-fs driving pulses. Given the slow evolution with increasing pulse duration, we expect that significant effects may still occur for much longer pulse durations (for which the computing time becomes prohibitive).

Considering the observed decrease of harmonic ellipticity when the pulses' durations shorten, we anticipate that harmonics generated with few-cycle pulses will strongly deviate from the perfect circularity. Obviously, in the extreme case where the HHG process is limited to 1/3 of ω cycle, the emitted attosecond pulse is linearly polarized [12].

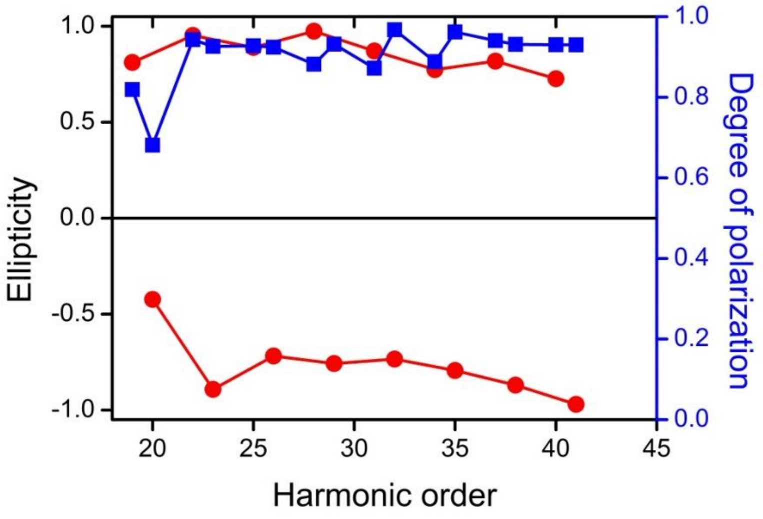

Supplementary Figure 6: Short-pulse envelope effect on the harmonic polarization. Ellipticity and degree of polarization calculated over the harmonic spectral width (+/- 0.25 order) for the interaction of 800 nm and 400 nm pulses of 10 ω-cycle FWHM envelope with a helium atom at $I_{\omega}=I_{2\omega}=2 \times 10^{14}$W/cm².

Supplementary Note 5: Influence of the relative phase of the two driving fields

We recall here the general expression of the electric field used in the simulations (see Methods section). The laser field is the sum of two counter rotating circularly polarized waves at λ_1 = 800 nm and λ_2 = 400 nm central wavelengths, respectively:

$$\boldsymbol{E(t)} = f(t)\{ [E_1 \cos(\omega_1 t + \varphi_{IR} + \varphi_{CEP}) + E_2 \cos(\omega_2 t + 2\,\varphi_{CEP})]\, \boldsymbol{e}_x + [E_1 \varepsilon_1 \sin(\omega_1 t + \varphi_{IR} + \varphi_{CEP}) + E_2\, \varepsilon_2 \sin(\omega_2 t + 2\,\varphi_{CEP})]\, \boldsymbol{e}_y \} \quad (9)$$

with $\omega_2 = 2 \times \omega_1$, $E_i = \frac{E_0}{\sqrt{1+\varepsilon_i^2}}$, and ε_i the field ellipticity, i = 1,2. φ_{CEP} is the carrier-envelope phase, acting on both ω_1 and ω_2 fields, whereas φ_{IR} is a phenomenological phase term applied only on the IR field for simulating a dephasing between the two fields induced, e.g., by instabilities in the Mach-Zehnder interferometer used to split the beams.

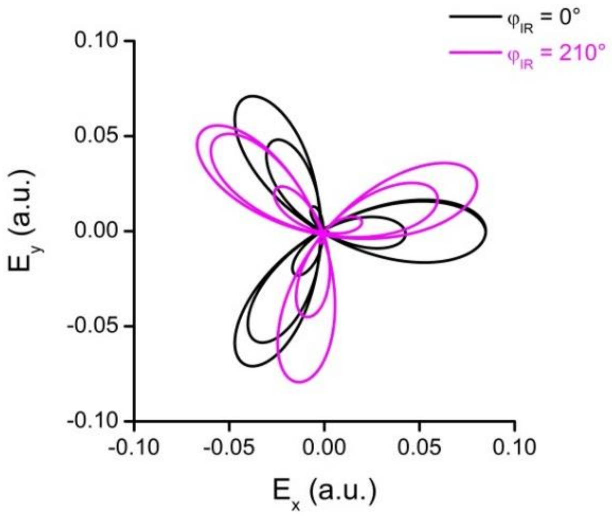

Supplementary Figure 7: Influence of the relative phase on the total field. Lissajous curves of the bicircular field for two $\omega - 2\omega$ relative phases φ_{IR} in the case of (ε_{ω} = +0.9, $\varepsilon_{2\omega}$ = -1) driving fields with trapezoidal envelopes.

In the case of constant pulse envelope and perfect circularity of the drivers, a change in the $\omega - 2\omega$ relative phase φ_{IR} (caused by, e.g., an instability of the two-color interferometer) simply rotates the total field rosette shape. The threefold dynamical symmetry therefore remains. When one of the drivers is elliptical, and *a fortiori* for short pulse envelopes, the dynamical symmetry is broken. The bicircular field evolution in the polarization plane then strongly depends on the $\omega - 2\omega$ relative phase φ_{IR}. Supplementary Fig. 7 shows the Lissajous curves of the total field between $t = 0$ and $t = 8T$ (end of the constant envelope region), for the trapezoidal envelopes f used in the calculations of main text Figs. 2 and 3. The construction of the total field over the 2-cycle turn-on is significantly different for the two relative phases, which has strong consequences when ionization confines efficient harmonic emission to the first cycles, as is the case in argon at 1.2 x 10^{14} W/cm^2.

The variations with φ_{IR} of the ellipticity ε and degree of polarization P for H16 and H17 generated in argon by such trapezoidal pulses are displayed in Supplementary Fig. 8. The ellipticity of both harmonics is strongly affected by a variation of φ_{IR}. The degree of polarization of H17 is more sensitive than the one of H16, as generally observed all along this study. In experiments where this relative phase is not controlled, long acquisition times result in an averaging over all the phase values. The consequence is a strong decrease of the degree of polarization of H17 to 61%, that of H16 remaining high at 93% (see Supplementary Table 2).

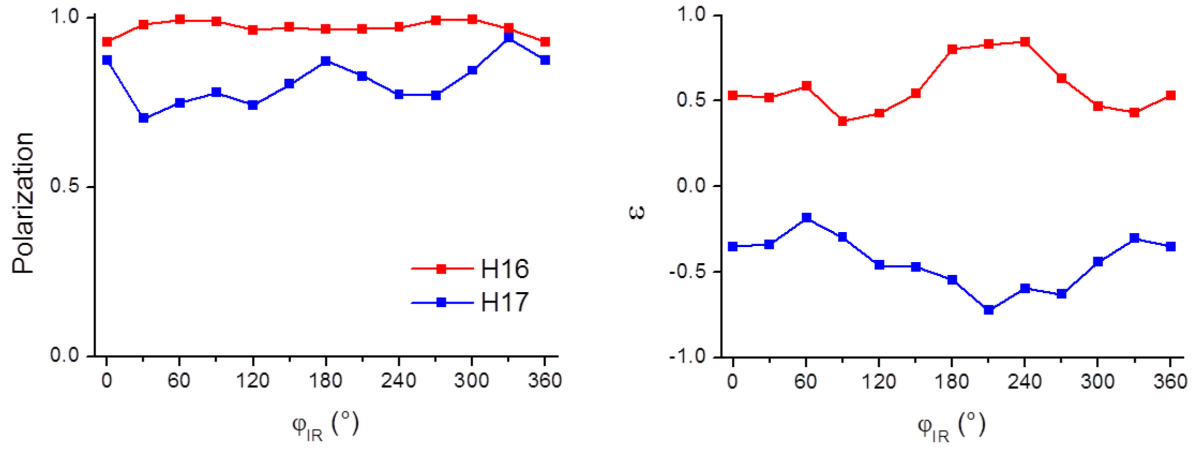

Supplementary Figure 8: Influence of the ω-2ω relative phase on the harmonic polarization. Degree of polarization (left) and ellipticity (right) of H16 and H17 calculated over the harmonic spectral width (+/- 0.25 order) generated in argon by trapezoidal bichromatic counter rotating (ε_{ω} = +0.9, $\varepsilon_{2\omega}$ = -1) pulses at an intensity $I_0=I_{\omega}=I_{2\omega}$= 1.2 x 10^{14} W/cm^2 as a function of the relative phase between the two pulses $\boldsymbol{\varphi_{IR}}$ (see Supplementary Eq. 9).

	ε H16	P H16	ε H17	P H17
$\varphi_{\mathrm{IR}} = 0$	+0.53	0.93	-0.35	0.87
$< \varphi_{\mathrm{IR}} >$ 0-360°	+0.60	0.93	-0.66	0.61

Supplementary Table 2: Consequence of the averaging over all ω-2ω relative phases. Comparison of the ellipticity and degree of polarization of H16 and H17 between two cases: i) $\varphi_{\mathrm{IR}} = 0$ and ii) a random averaging over all φ_{IR} values.

Supplementary Note 6 : Simple polarization model in the case of an exponentially decreasing efficiency

In this simple model, the total harmonic electric field is written as the sum of linearly polarized δ-like fields, emitted every $T/3$ with a polarization angle changing by 120° every $T/3$.

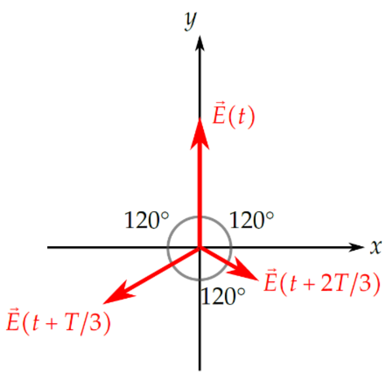

The field amplitude E is considered proportional to the number of emitters in the medium, which is exponentially decreasing with the characteristic lifetime of the dipoles in main text Fig. 2a:

$$E(t) \propto e^{-t/\tau} \tag{10}$$

The qth harmonic emissions along the x and y components are then written as:

$$A_x^{(q)}(t) = \frac{\sqrt{3}}{2}\left[E\left(t-\frac{2T}{3}\right)\cdot\delta\left(t-\frac{2T}{3}\right) - E\left(t-\frac{T}{3}\right)\cdot\delta\left(t-\frac{T}{3}\right)\right]e^{iq\omega t} \tag{11}$$

$$A_y^{(q)}(t) = \left[E(t)\cdot\delta(t) - \frac{1}{2}E\left(t-\frac{T}{3}\right)\cdot\delta\left(t-\frac{T}{3}\right) - \frac{1}{2}E\left(t-\frac{2T}{3}\right)\cdot\delta\left(t-\frac{2T}{3}\right)\right]e^{iq\omega t} \tag{12}$$

In argon at an intensity of 8 x 10^{13} W/cm², the dipole decreases exponentially with a characteristic time of $\tau(8\ \times 10^{13}) = 2.5\,T$. The resulting ellipticity is $|\varepsilon| = 0.86$, identical for all harmonic orders. When the intensity is increased to 1.2 x 10^{14} W/cm², the decay of the dipole due to ionization of the system is even faster ($\tau(1.2\ \times 10^{14}) = 1.3\,T$), and the ellipticity is decreased to $|\varepsilon| = 0.74$. Note that, with an exponential decay of the dipole, the amplitude ratios of the three consecutive linearly polarized pulses of the harmonic emission remain the same across the pulse duration. Thus, the polarization characteristics do not vary temporally in this model, and the harmonics remain fully polarized ($P = 1$).

Supplementary Note 7 : Discussion of the shape of the harmonic spectra

The relative intensity of the different harmonic orders contains a wealth of information on the generation process. First, the intensity of the 3q orders may reveal a breaking of the dynamical symmetry of the interaction, see e.g. [13, 14]. Second, the intensity ratio between the 3q+1 and the 3q+2 orders may provide information on phase matching [8, 15], on the electronic structure of the generation medium [12, 16, 17] or on the intensity [18] and overlap [12] of the driving fields.

For the experimental spectra displayed in main text Fig. 5, we observe in (a) and (b), that is, for ($\varepsilon_\omega \approx$ -1, $\varepsilon_{2\omega} \approx$ +1) and ($\varepsilon_\omega \approx$ +1, $\varepsilon_{2\omega} \approx$ -1) resp., a strong attenuation of H15 (3q) and a favored population of H16 (3q+1) relative to H17 (3q+2), e.g. for (a) H15/H16 ≈ 0.1 and H17/H16 ≈ 0.42. The slight differences in the spectra in (a) and (b) is attributed to a small deviation from circularity of the drivers induced by imperfect finite-bandwidth quarter wave-plates [13].

When the helicity of the ω field is reduced ($\varepsilon_\omega \approx$ +0.84, $\varepsilon_{2\omega} \approx$ -1), a significant increase of H15 is observed in (**c**) while the H17/H16 ratio increases: H15/H16 ≈ 0.7 and H17/H16 ≈ 0.65.

While the origin of the increased H15 intensity is clear (see simulations in main text Fig. 3), further experimental and theoretical investigations are needed to determine unambiguously the origin of

the variation of the H17/H16 ratio among the different effects mentioned above. Finally, we stress that discussing the ellipticity of the XUV emission only based on the relative spectral intensity of the components co-rotating or counter-rotating with the ω field (as shown in main text Fig. 1d) may be misleading. Indeed, the degree of circular polarization, calculated from the intensities of the left- and right- circular components of the light, writes:

$$s_3 = \frac{I_R - I_L}{I_R + I_L} = \frac{1-\dfrac{I_L}{I_R}}{1+\dfrac{I_L}{I_R}} \tag{13}$$

On the other hand, the ellipticity writes:

$$\varepsilon = \tan\left[\frac{1}{2}\sin^{-1}\left(\frac{s_3}{\sqrt{s_1^2+s_2^2+s_3^2}}\right)\right] \tag{14}$$

If the light is fully polarized, Supplementary Eqs. (13) and (14) show that a 2-order-of-magnitude difference between the intensities of the left- and right- circular components of the light only gives $\varepsilon = 0.8$, which is far from full circularity. A 4-order-of-magnitude difference is necessary to achieve $\varepsilon = 0.98$.

Supplementary references

[1] Lebech, M. et al. Complete description of linear molecule photoionization achieved by vector correlations using the light of a single circular polarization. *J. Chem. Phys.* **118**(21), 9653–9663 (2003).

[2] Born, M. & Wolf, E. *Principles of Optics (Sixth Edition)*. Cambridge University Press, (1980).

[3] Dowek, D. & Lucchese, R. R. Photoionization dynamics: photoemission in the molecular frame of small molecules ionized by linearly and elliptically polarized light. In *Dynamical Processes in Atomic and Molecular Physics*, 57–95. Bentham Science Publishers (2012).

[4] Veyrinas, K. et al. Complete determination of the state of elliptically polarized light by electron-ion vector correlations. *Phys. Rev. A* **88**(6), 063411 (2013).

[5] Lucchese, R. R. et al. Polar and azimuthal dependence of the molecular frame photoelectron angular distributions of spatially oriented linear molecules. *Phys. Rev. A* **65**, 020702 (2002).

[6] Veyrinas, K. *Photoémission dans le référentiel moléculaire : une sonde de la dynamique électronique et nucléaire et de l'état de polarisation du rayonnement ionisant*. Ph. D. thesis, Université Paris Sud - Paris XI, (2015).

[7] Fleischer, A., Kfir, O., Diskin, T., Sidorenko, P., & Cohen, O. Spin angular momentum and tunable polarization in high-harmonic generation. *Nature Photonics* **8**(7), 543–549 (2014).

[8] Kfir, O. et al. Generation of bright phase-matched circularly-polarized extreme ultraviolet high harmonics. *Nature Photonics* **9**(2), 99–105 (2015).

[9] Jiménez-Galán, A., Zhavoronkov, N., Schloz, M., Morales, F., & Ivanov, M. Time-resolved high harmonic spectroscopy of dynamical symmetry breaking in bi-circular laser fields: the role of Rydberg states. *Opt. Expr.* **25**(19), 22880–22896 (2017).

[10] Veyrinas, K. et al. Molecular frame photoemission by a comb of elliptical high-order harmonics: a sensitive probe of both photodynamics and harmonic complete polarization state. *Faraday Discuss.* **194**, 161–183 (2016).

[11] Hecht, E. *Optics (Fourth Edition)*. Addison Wesley, (2001).

[12] Medišauskas, L., Wragg, J., Hart, H., & Ivanov, M. Generating isolated elliptically polarized attosecond pulses using bichromatic counterrotating circularly polarized laser fields. *Phys. Rev. Lett.* **115**(15), 153001 (2015).

[13] Fan, T. et al. Bright circularly polarized soft X-ray high harmonics for X-ray magnetic circular dichroism. *PNAS* **112**(46), 14206–14211 (2015).

[14] Baykusheva, D., Ahsan, M., Lin, N., & Wörner, H. Bicircular High-Harmonic spectroscopy reveals dynamical symmetries of atoms and molecules. *Phys. Rev. Lett.* **116**(12), 123001 (2016).

[15] Zhavoronkov, N. & Ivanov, M. Extended ellipticity control for attosecond pulses by high harmonic generation. *Opt. Lett.* **42**(22), 4720–4723 (2017).

[16] Miloševic, D. B. Circularly polarized high harmonics generated by a bicircular field from inert atomic gases in the p state: A tool for exploring chirality-sensitive processes. *Phys. Rev. A* **92**(4), 043827 (2015).

[17] Baykusheva, D., Brennecke, S., Lein, M., & Wörner, H. J. Signatures of electronic structure in bicircular high-harmonic spectroscopy. *Phys. Rev. Lett.* **119**, 203201 (2017).

[18] Dorney, K. M. et al. Helicity-selective enhancement and polarization control of attosecond high harmonic waveforms driven by bichromatic circularly polarized laser fields. *Phys. Rev. Lett.* **119**, 063201 (2017).
